# Identification of a major IP_5_ kinase in *Cryptococcus neoformans* confirms that PP-IP_5_/IP_7_, not IP_6_, is essential for virulence

**DOI:** 10.1038/srep23927

**Published:** 2016-04-01

**Authors:** Cecilia Li, Sophie Lev, Adolfo Saiardi, Desmarini Desmarini, Tania C. Sorrell, Julianne T. Djordjevic

**Affiliations:** 1 Centre for Infectious Diseases and Microbiology, The Westmead Institute for Medical Research, The University of Sydney, Westmead, NSW, Australia; 2 Medical Research Council Laboratory for Molecular Cell Biology, University College London, London, UK; 3 Marie Bashir Institute for Infectious Diseases and Biosecurity, University of Sydney, NSW, Australia; 4 Westmead Hospital, Westmead, NSW, Australia

## Abstract

Fungal inositol polyphosphate (IP) kinases catalyse phosphorylation of IP_3_ to inositol pyrophosphate, PP-IP_5_/IP_7_, which is essential for virulence of *Cryptococcus neoformans*. Cryptococcal Kcs1 converts IP_6_ to PP-IP_5_/IP_7_, but the kinase converting IP_5_ to IP_6_ is unknown. Deletion of a putative IP_5_ kinase-encoding gene (*IPK1*) alone (*ipk1*Δ), and in combination with *KCS1* (*ipk1*Δ*kcs1*Δ), profoundly reduced virulence in mice. However, deletion of *KCS1* and *IPK1* had a greater impact on virulence attenuation than that of *IPK1* alone. *ipk1*Δ*kcs1*Δ and *kcs1*Δ lung burdens were also lower than those of *ipk1*Δ. Unlike *ipk1*Δ, *ipk1*Δ*kcs1*Δ and *kcs1*Δ failed to disseminate to the brain. IP profiling confirmed Ipk1 as the major IP_5_ kinase in *C. neoformans*: *ipk1*Δ produced no IP_6_ or PP-IP_5_/IP_7_ and, in contrast to *ipk1*Δ*kcs1*Δ, accumulated IP_5_ and its pyrophosphorylated PP-IP_4_ derivative. Kcs1 is therefore a dual specificity (IP_5_ and IP_6_) kinase producing PP-IP_4_ and PP-IP_5_/IP_7_. All mutants were similarly attenuated in virulence phenotypes including laccase, urease and growth under oxidative/nitrosative stress. Alternative carbon source utilisation was also reduced significantly in all mutants except *ipk1*Δ, suggesting that PP-IP_4_ partially compensates for absent PP-IP_5_/IP_7_ in *ipk1*Δ grown under this condition. In conclusion, PP-IP_5_/IP_7_, not IP_6_, is essential for fungal virulence.

The fungal pathogen*, Cryptococcus neoformans*, predominantly infects immunocompromised individuals via the lung and then disseminates to the brain where it establishes life-threatening meningoencephalitis. *C. neoformans* is responsible for over half a million deaths each year in AIDS patients alone^1^. Some of the most well-known virulence factors of *C. neoformans* include a polysaccharide capsule^2^, melanin^3^ and urease^4^,^5^. A prerequisite to virulence is its ability to grow at human physiological temperature, which can impact the stability of the cell wall^6^,^7^. In the host, *C. neoformans* also encounters oxidative and nitrosative stress, which originate predominantly from macrophages and altered nutritional availability in the lung and brain, which are low in glucose^8^,^9^,^10^,^11^,^12^,^13^.

Several signalling cascades, including calcineurin, mitogen-activated protein kinase/protein kinase C (Mpk1/Pkc1), cyclic adenosine monophosphate/protein kinase A (cAMP/Pka1), high osmolarity glycerol (HOG), and Rim101 pathways, allow *C. neoformans* to sense, respond and adapt to host stresses encountered throughout the course of infection^14^,^15^,^16^,^17^,^18^,^19^,^20^. We previously identified a new virulence-related signalling pathway in *C. neoformans* comprising phospholipase C1 (Plc1) and a series of sequentially acting inositol polyphosphate kinases (IPKs)^21^,^22^,^23^. The IPKs convert the inositol trisphosphate (IP_3_) product of Plc1 to IP_4_-IP_6_, and IP_6_ to the inositol pyrophosphates, PP-IP_5_/IP_7_ and (PP)_2_-IP_4_/IP_8_. Specifically, IP_3_ is converted to IP_4_ and IP_5_ by Arg1^22^,^23^. An uncharacterised kinase then phosphorylates IP_5_ to produce the highly abundant IP_6_ species. IP_6_ is then phosphorylated by Kcs1 to PP-IP_5_/IP_7_. Asp1 further phosphorylates PP-IP_5_/IP_7_ to produce (PP)_2_-IP_4_/IP_8_^23^. *ARG1* and *KCS1* deletion mutants of *C. neoformans*, which do not produce PP-IP_5_/IP_7_ and (PP)_2_-IP_4_/IP_8_, exhibit attenuated growth, compromised cell wall integrity and reduced production of melanin, urease and mating filaments. The *KCS1* deletion strain, *kcs1*Δ, is also unable to utilise alternative carbon sources for growth^23^. Consequently *kcs1*Δ has a reduced ability to infect host lung, does not disseminate to the brain and is avirulent in a mouse model^23^. However, (PP)_2_-IP_4_/IP_8_ plays an insignificant role in cryptococcal virulence *per se*, since the IP_8_-deficient mutant strain, *asp1*Δ, had a similar virulence profile to that of the wild-type strain^23^. Our results therefore support a crucial role for PP-IP_5_/IP_7_ in the pathogenicity of *C. neoformans*^23^. While IP_6_ is the precursor for the synthesis of virulence-promoting PP-IP_5_/IP_7_ in *C. neoformans,* neither the IP_5_ kinase responsible for production of IP_6_, nor the contribution of IP_6_ to fungal pathogenicity is currently known.

In this study we employ combinatorial gene deletion analysis, HPLC-based inositol polyphosphate profiling, phenotypic analysis and a mouse infection model to establish Ipk1 as the major IP_5_ kinase in *C. neoformans*, and assess the contribution of the Ipk1 product, IP_6_, to pathogenicity. Similar to Kcs1 we found that Ipk1 is essential for pathogenicity but that its contribution relates more to its indirect role in PP-IP_5_/IP_7_ production, rather than its direct role in producing IP_6_. Our results therefore confirm that PP-IP_5_/IP_7_ is the most crucial IP species for cryptococcal pathogenicity and that the contribution of IP_6_ is relatively insignificant.

## Results

### Identification of an IP_5_ kinase in *C. neoformans*

#### Homology search

We recently identified Arg1 as the major IP_3_ kinase in *C. neoformans* (*Cn*) converting Plc1-derived IP_3_ to IP_5_^22^,^23^. We also identified Kcs1 as the major cryptococcal IP_6_ kinase, phosphorylating IP_6_ to the inositol pyrophosphate PP-IP_5_/IP_7_, which is crucial for cryptococcal virulence^23^. To identify the intermediary kinase in the pathway, we searched the *C. neoformans var grubii* (strain H99) genomic database (http://www.broadinstitute.org/annotation/genome/cryptococcus_neoformans/MultiHome.html) for an IP_5_ kinase homolog using Ipk1 from *Saccharomyces cerevisiae* as a query. CNAG_01294 produced the strongest match and was designated *CnIPK1. CnIPK1* is 3245 nucleotides in length and is predicted to encode a protein of 415 amino acids. Using the global alignment sequence analysis tool available from https://npsa-prabi.ibcp.fr/cgi-bin/npsa_automat.pl?page=/NPSA/npsa_clustalw.html, the *Cn*Ipk1 protein was found to be just 13.7% identical and 26.68% similar to *Sc*Ipk1, and 19.18% identical and 28.96% similar to human IP_5_ kinase, inositol-pentakisphosphate 2-kinase. Despite the low homology, a domain search at (http://pfam.xfam.org/) confirmed the presence of the inositol-pentakisphosphate 2-kinase protein domain (PF06090) at the N-terminus of *CnIPK1*.

#### Creation of IPK1 deletion mutant strains

To determine whether *Cn*Ipk1 is an IP_5_ kinase that produces IP_6_, an *IPK1* deletion mutant (*ipk1*Δ) and an *IPK1* reconstituted strain (*ipk1*Δ + *IPK1*) were created in the wild-type H99 (WT H99) background using biolistic transformation and homologous recombination as described in the methods. *KCS1* was also deleted in *ipk1*Δ to create an *ipk1*Δ *kcs1*Δ double mutant. Targeted gene deletion and genetic reconstitution were confirmed by PCR and antibiotic resistance testing (see supplementary methods and Supplementary Fig. S2).

#### Comparison of IP profiles of deletion mutants using inositol radiolabelling and HPLC

**Figure 1:**
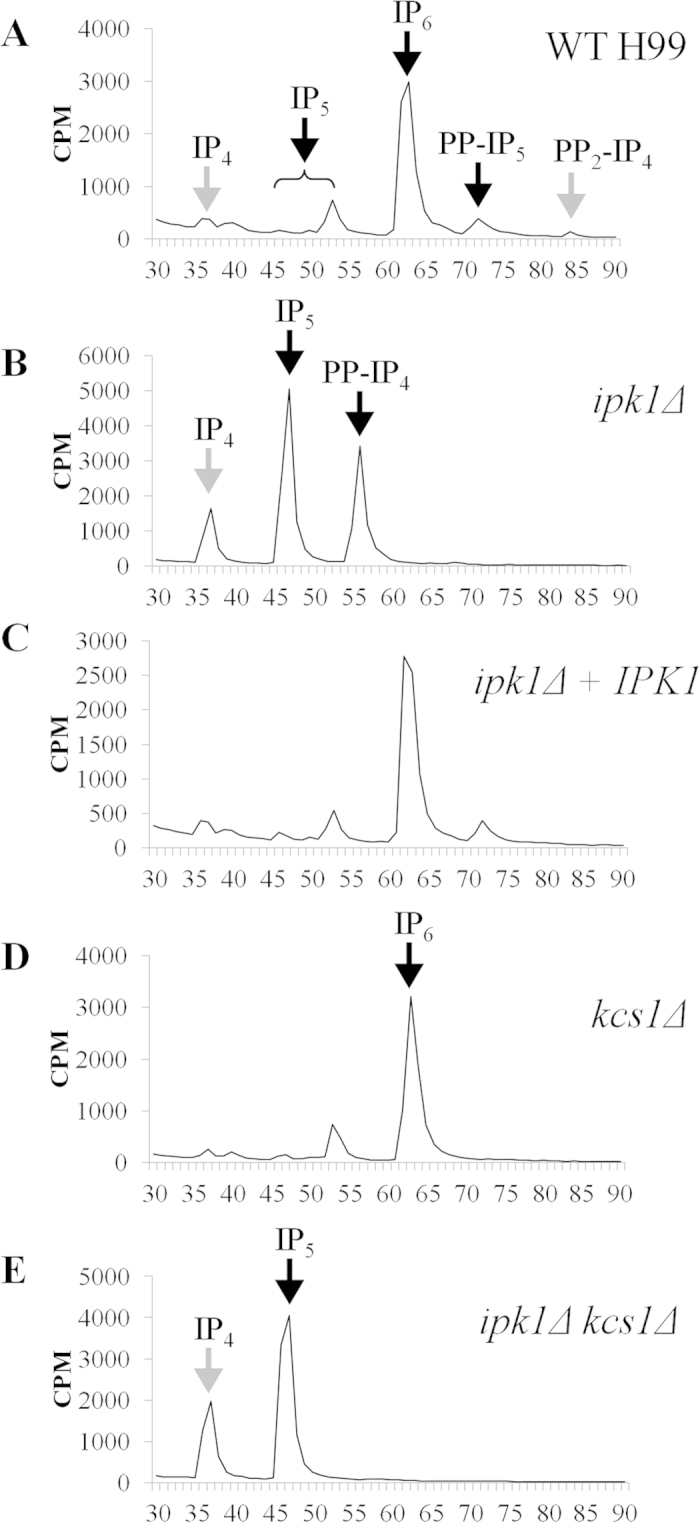
IP profiles of (**A**) WT, (**B**) *ipk1*Δ, (**C**) *ipk1*Δ + *IPK1*, (**D**) *kcs1*Δ and (**E**) *ipk1*Δ *kcs1*Δ. Lysates prepared from [^3^H] myo-inositol-labelled cells were subjected to anion-exchange HPLC analysis. IP species were eluted using a gradient of increasing phosphate concentration. The elution profile of the IP standards used (IP_5_ isomer [^3^H]I(1, 3, 4, 5, 6)P_5_, [^3^H]IP_6_, [^3^H]PP-IP_4_ and [^3^H]PP-IP_5_/IP_7_) are indicated by the black arrows; grey arrows indicate the expected elution profile of I(1, 3, 4, 5)P_4_ and (PP)_2_-IP_4_/IP_8_ species.

WT, *ipk1*Δ, *ipk1*Δ + *IPK1* and the *kcs1*Δ strains were radiolabelled with [^3^H] myo-inositol. The IP profile of lysates prepared from each strain was then compared by anion-exchange HPLC (Fig. 1). Similar to many eukaryotic cells including fungi, plants and humans, IP_6_ was found to be the most abundant IP species in the WT cryptococcal strain (Fig. 1A). PP-IP_5_/IP_7_ and (PP)_2_-IP_4_/IP_8_ were detected in the WT strain, but not in *kcs1*Δ control strain as reported previously^23^. In contrast, IP_6_, PP-IP_5_/IP_7_ and (PP)_2_-IP_4_/IP_8_ were not detected in *ipk1*Δ, while IP_5_ and IP_4_ were increased (Fig. 1B). These results are consistent with Ipk1 being the major IP_5_ kinase in *C. neoformans.* The reintroduction of an intact *IPK1* gene into *ipk1*Δ restored IP_5_ kinase activity back to the level of the WT strain (Fig. 1C). In addition to IP_4_ and IP_5_, an IP species with an elution time corresponding to PP-IP_4_^24^ accumulated in the *ipk1*Δ mutant (Fig. 1B) but was absent in the profiles of WT and *kcs1*Δ (Fig. 1A,D). To determine whether the cryptococcal IP_6_ kinase, Kcs1, recognises IP_5_ as an alternative substrate to IP_6_ in the *ipk1*Δ mutant, and converts it to PP-IP_4_, the IP profile of the double *ipk1*Δ *kcs1*Δ gene deletion mutant was assessed. We hypothesised that PP-IP_4_ would not be detected in the double mutant if Kcs1 is the progenitor of PP-IP_4_. The profile of *ipk1*Δ *kcs1*Δ (Fig. 1E) confirms this prediction and indicates that Kcs1 synthesises the pyrophosphate-containing PP-IP_4_ using IP_5_ as substrate. However, the absence of PP-IP_4_ in the WT and *kcs1*Δ strains, which both have active Ipk1, indicates that Kcs1 acts predominantly as an IP_6_ kinase when Ipk1 is present.

### Impact of Ipk1 on stress tolerance and production of virulence traits

**Figure 2:**
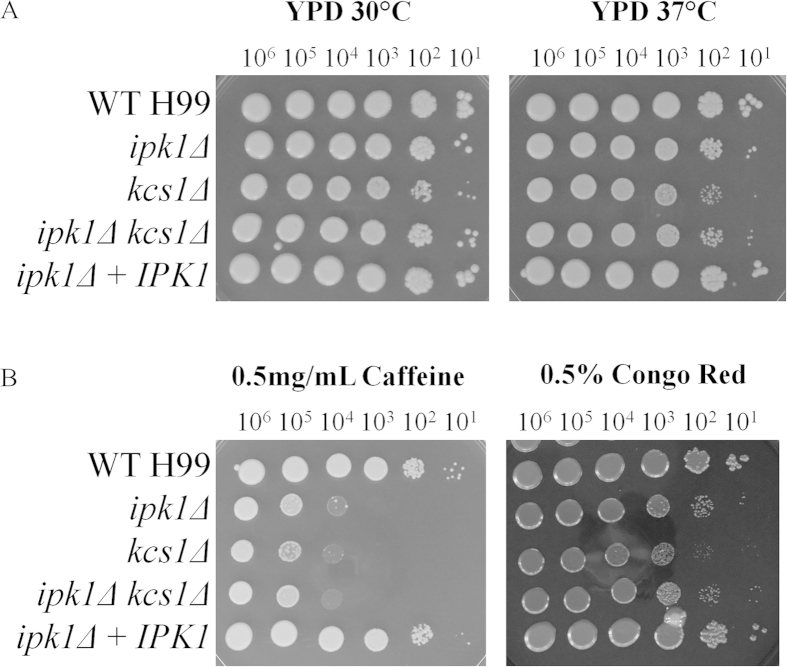
Effect of temperature (**A**) and cell wall perturbing agents (**B**) on growth of *ipk1*Δ, *kcs1*Δ and *ipk1*Δ *kcs1*Δ. All strains were serially diluted 10-fold, from 10^6^ cells to 10^1^ cells per 3μL (left to right) and dropped onto media containing the reagents indicated. All plates were incubated at 30 °C/37 °C for 72 hours. The YPD 30 °C plate was used as a control.

**Figure 3:**
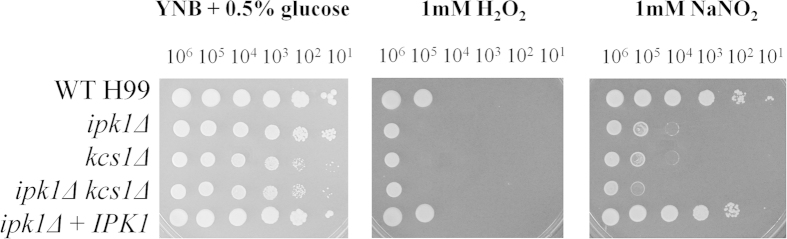
Effect of oxidative and nitrosative stress on growth of *ipk1*Δ, *kcs1*Δ and *ipk1*Δ*kcs1*Δ. All strains were serially diluted 10-fold, from 10^6^ cells to 10^1^ cells per 5μL (left to right) and dropped onto YNB + 0.5% glucose agar containing the reagents indicated. All plates were incubated at 37 °C for 96 hours.

We recently demonstrated that the PP-IP_5_/IP_7_ produced by Kcs1 is crucial for growth of *C. neoformans* under stress: the *kcs1*Δ mutant exhibited reduced growth in the presence of cell wall perturbing agents, and reduced production of the virulence factors, melanin and urease^23^. While PP-IP_5_/IP_7_ is absent in the *kcs1*Δ, *ipk1*Δ and *ipk1*Δ *kcs1*Δ mutants, the *ipk1*Δ and *ipk1*Δ *kcs1*Δ mutants also fail to produce IP_6_ (see Table 1 for a summary). We therefore investigated whether *ipk1*Δ and *ipk1*Δ *kcs1*Δ exhibit these growth and virulence phenotypes and whether they are attenuated to a greater extent than *kcs1*Δ due to the absence of both IP_6_ and PP-IP_5_/IP_7_. As *C. neoformans* can replicate inside macrophages, tolerance of oxidative and nitrosative stress was also investigated. The results show that the *kcs1*Δ mutant, but not the *ipk1*Δ mutant, displayed a mild growth defect on rich medium (YPD) at 30 °C and 37 °C (Fig. 2A). However, the *ipk1*Δ mutant exhibited a significant growth defect in the presence of two cell wall perturbing agents, Congo red and, (even more so), caffeine (Fig. 2B). All mutant strains were mildly sensitive to oxidative stress at 37 °C and strongly sensitive to nitrosative stress (Fig. 3). In contrast to our expectation, the *ipk1*Δ growth defect under all of the conditions tested (Figs 2 and 3) was similar to, or mildly better, than that observed for the *kcs1*Δ and *ipk1*Δ *kcs1*Δ mutant strains, indicating that loss of IP_6_ in combination with PP-IP_5_/IP_7_ does not exacerbate the phenotype. Growth of *ipk1*Δ + *IPK1* was similar to WT under all stress conditions tested.

#### Production of virulence traits

**Figure 4:**
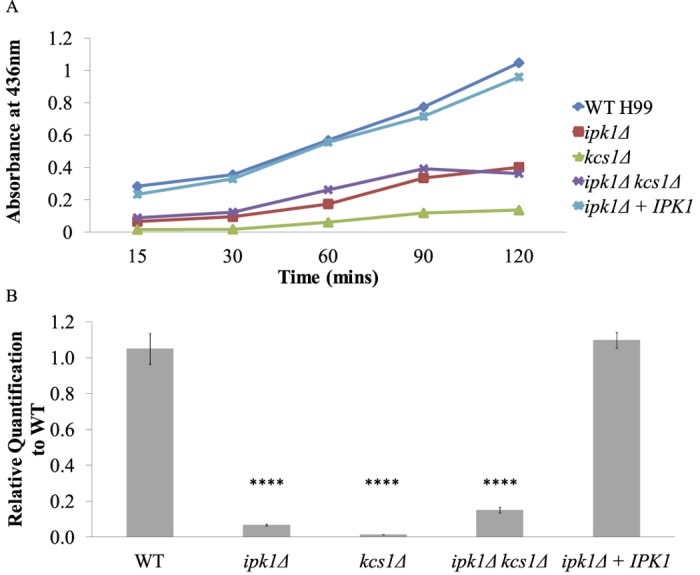
Effect of IPK gene deletion on extracellular laccase activity (**A**) and *LAC1* gene expression (**B**). Strains were incubated for 3 and 6 hours at 30 °C in minimal media without glucose for gene induction and enzyme assay, respectively. (**A**) ABTS was then added and the amount of oxidised ABTS at each time point was measured spectrophotometrically at 436 nm. (**B**) *LAC1* gene expression was determined by qRT-PCR with *ACT1* used as the reference gene for normalization. A one-way ANOVA multiple comparisons test of all strains revealed a statistically significant difference between the 3 mutants and both WT and *ipk1*Δ + *IPK1*. (****p-value < 0.0001 and error bars represent standard deviation).

**Figure 5:**
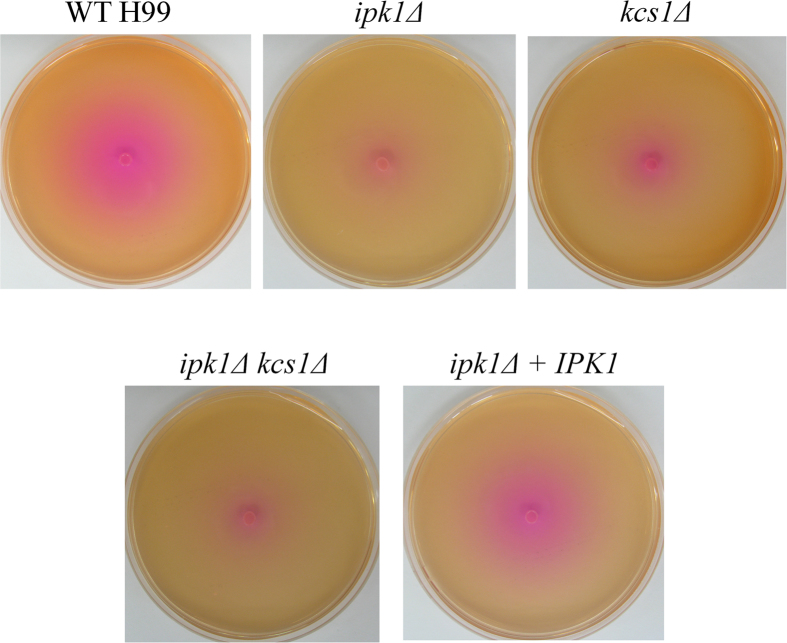
Effect of IPK gene deletion on urease production 10^6^ cells of each strain was dropped onto Christensen’s (urea-containing) agar. All plates were incubated at 30 °C for 72 hours. The extent of urease production, which is observed due to the incorporation of the phenol red pH indicator, is proportional to the diameter of the pink halo surrounding the inoculum.

The *kcs1*Δ mutant exhibits defects in laccase 1 (Lac1)-induced melanisation: in a glucose-deficient environment, expression of the Lac1-encoding gene, *LAC1*, is repressed in *kcs1*Δ^23^. We therefore investigated whether the *IPK1*-deficient mutants displayed a similar phenotype. Since laccase is cell wall-associated, extracellular laccase activity was quantified by measuring the oxidation of ABTS by whole cells over 120 min following 6 hours of induction in glucose-deficient medium (Fig. 4A). The induction of *LAC1* mRNA after 3 hours induction in glucose-deficient medium was also measured by qRT-PCR (Fig. 4B). Relative to WT and *ipk1*Δ + *IPK1*, cell-associated laccase activity was reduced in all mutants (Fig. 4A). This reduction in ABTS oxidation correlated with reduced *LAC1* mRNA expression (Fig. 4B). Similarly, urease production was reduced in all deletion mutants compared to WT and *ipk1*Δ + *IPK1* as indicated by the diameter of the pink halo surrounding the inoculum, following growth on Christensen’s agar (Fig. 5).

#### IPK mutants are hypersusceptible to antifungal drugs

Since the *arg1*Δ and *kcs1*Δ mutants are hypersusceptible to antifungal drugs^23^ we investigated the susceptibility of the *ipk1*Δ and *ipk1*Δ *kcs1*Δ mutants to a range of clinically-available antifungals, and included *kcs1*Δ as a control (Supplementary Table S1). Similar to WT and *kcs1*Δ, *ipk1*Δ and *ipk1*Δ *kcs1*Δ retained their resistance to the echinocandins: anidulafundin, micafungin and caspofungin. Collectively, the mutants were 2–4 times more sensitive to 5-flucytosine and 4–8 times more sensitive to the azole family (posaconazole, voriconazole, itraconazole and fluconazole). The sensitivity of all mutants to amphotericin B was similar to WT.

#### Growth on alternative carbon sources

**Figure 6:**
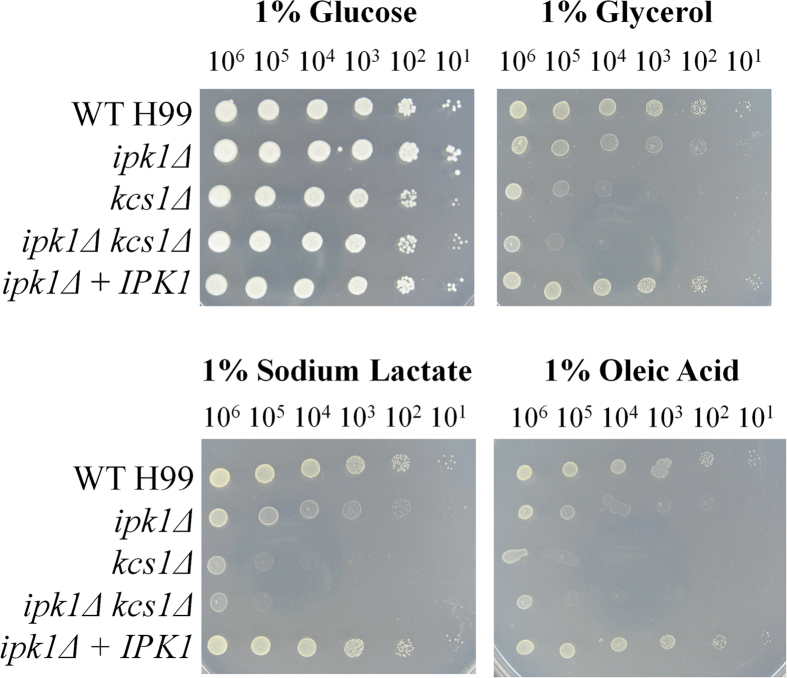
Effect of *IPK1* deletion on alternative carbon source utilisation. All strains were serially-diluted 10-fold, from 10^6^ cells to 10^1^ cells per 3 μL (left to right) and dropped onto minimal media (MM) containing the carbon sources indicated. MM +1% glucose was used as a control. All plates were incubated at 30 °C for 72 hours.

**Figure 7:**
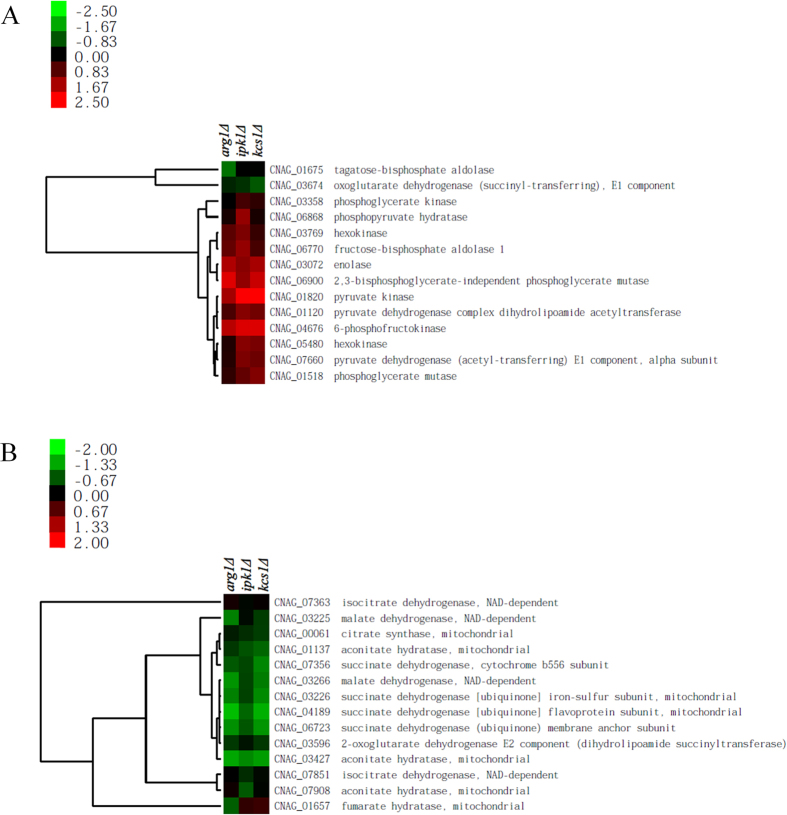
Expression of glycolysis (**A**) and TCA cycle (**B**) associated genes in *arg1*Δ, *ipk1*Δ and *kcs1*Δ mutants, relative to WT H99. Wild type and mutant cultures grown overnight in YPD broth were used for RNA extraction, followed by RNA-seq gene expression analysis (green, down-regulated expression; red, up-regulated expression). The colour bar to the left of each heat map demonstrates the log_2_ fold changes from comparison of each mutant to WT.

Pathogens of the respiratory system including *C. neoformans* encounter a low glucose environment during host lung infection^9^ and cryptococcal metabolic mutants incapable of utilising carbon sources other than glucose are attenuated for virulence in animal models^25^,^26^. We previously demonstrated that the PP-IP_5_/IP_7_-deficient *kcs1*Δ strain is impaired in utilising three non-fermentable carbon sources: glycerol, lactate, and oleic acid. We therefore compared the growth of the *IPK1*-deficient mutant strains to that of *kcs1*Δ when these substrates are used as the sole carbon source (Fig. 6). On the control plate containing glucose, all mutant strains grew at a similar rate to that of WT and *ipk1*Δ + *IPK1*. However, on plates containing glycerol, lactate or oleic acid, growth of all of the mutants was reduced. Unlike growth phenotypes in Figs 2 and 3, growth of *kcs1*Δ and *ipk1*Δ *kcs1*Δ on these alternative carbon sources was more severely attenuated than growth of the *ipk1*Δ mutant. The growth defect seen in *kcs1*Δ correlated with RNA-seq analysis where expression of genes involved in the utilisation of alternative carbon sources was reduced^23^. We used the same approach to compare the gene expression profile of *ipk1*Δ with those of *kcs1*Δ and *arg1*Δ. Arg1 encodes the major IP_3_ kinase in *C. neoformans* and, like *ipk1*Δ and *kcs1*Δ, *arg1*Δ does not produce PP-IP_5_/IP_7_. Figure 7 provides a representative summary of the expression of genes associated with glycolysis (Fig. 7A) and the tricarboxylic acid (TCA) cycle (Fig. 7B). Glycolysis-related genes in Fig. 7A were similarly up-regulated in *arg1*Δ, *ipk1*Δ and *kcs1*Δ, relative to WT. However, although genes encoding TCA cycle enzymes were down-regulated in all mutants, they were down-regulated to a lesser extent in *ipk1*Δ, compared with other mutants (Fig. 7B). The expression of genes involved in fatty acid β-oxidation and peroxisomal organisation was similarly down-regulated in all mutants (Supplementary Fig. S1).

### Impact of Ipk1 on cryptococcal virulence in a mouse model

**Figure 8:**
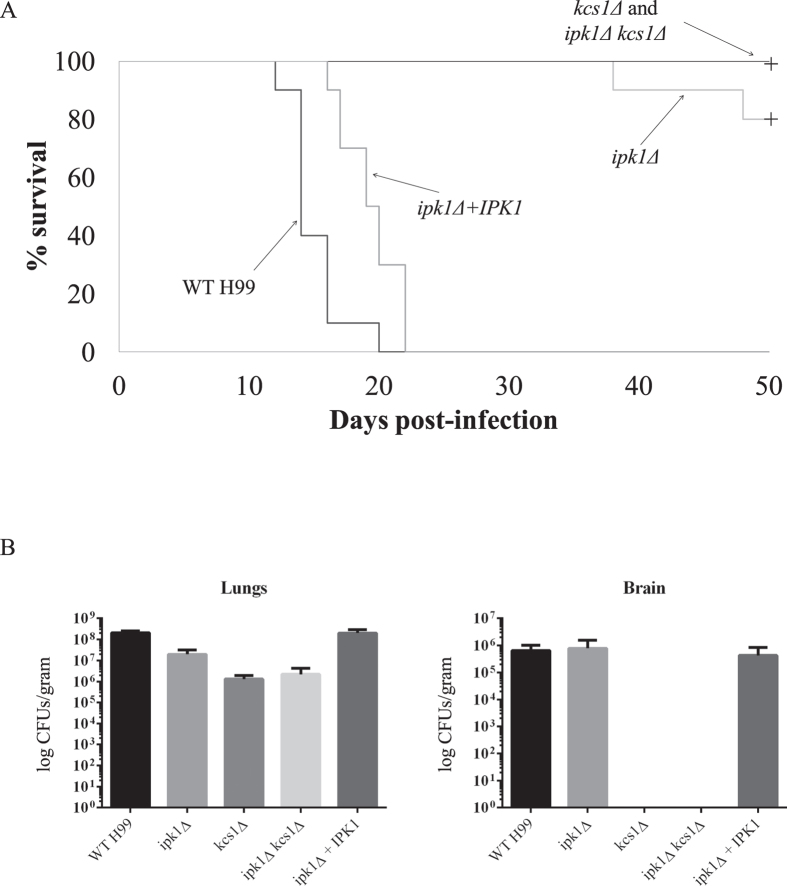
Effect of IPK deletion on virulence in a murine inhalation model of cryptococcosis. (**A**) Survival analysis. Isoflurane-anaesthetised mice were inoculated intranasally with 5 × 10^5^ CFUs of WT, *ipk1*Δ, *kcs1*Δ, *ipk1*Δ *kcs1*Δ and *ipk1*Δ+*IPK1*, and euthanised after showing debilitating symptoms of infection, or at 50 days post-infection for asymptomatic mice (see Methods). A survival analysis (Kaplan-Meier log rank test) revealed statistically significant differences in survival between the mutant groups and both WT and *ipk1*Δ+*IPK1* (*p* < 0.001). Median survival of mice infected with WT H99 and *ipk1*Δ+*IPK1* was 14 and 19 days, respectively, but the difference was not statistically significant (*p* > 0.05). Note that similar to *kcs1*Δ-infected mice^23^ all *ipk1*Δ *kcs1*Δ-infected mice were healthy with no sign of illness or weight loss for up to at least 50 days post-infection. (**B**) Organ burden analysis. Infection burdens were determined at time of death or upon termination of the experiment (Day 50). For each strain, organs were harvested from 3 mice. Results represent the mean logCFUs ± SEM. The PP-IP_4_-deficient strains, *kcs1*Δ and *ipk1*Δ *kcs1*Δ, did not disseminate to the brain.

We found previously that the *kcs1*Δ mutant is avirulent in a murine inhalational model of cryptococcosis but establishes a persistent asymptomatic infection that remains confined to the lungs for up to 50 days post infection^23^. Thus, we compared the infection profile of the IPK deletion mutants using the same model. Mice were inoculated with 5 × 10^5^ CFUs of WT, *ipk1*Δ, *ipk1*Δ *kcs1*Δ and *ipk1*Δ + *IPK1*, and time to illness (survival) and organ burdens were determined (Fig. 8A). The results for *kcs1*Δ-infected mice were also included in the analysis as a comparison. All WT- and *ipk1*Δ + *IPK1*-infected mice succumbed to infection over a similar time period (median survival time was 14 and 19 days, respectively; *p* = 0.264). However, 80% and 100% of *ipk1*Δ- and *ipk1*Δ *kcs1*Δ*-*infected mice, respectively, survived the infection and maintained their weight and vigour over the 50 day time course. The differences in survival between the 3 mutant-infected groups and the WT- and *ipk1*Δ+*IPK1-*infected groups was statistically significant (*p*-value < 0.001) as determined by the Kaplan-Meier log rank test.

Lung and brain infection burdens for the WT- and *ipk1*Δ + *IPK1*- infected groups (at time of death) and the mutant-infected groups (at time of death or 50 days post-infection for still healthy mice) were also measured (Fig. 8B). Lower lung burdens were obtained for all of the mutant strains relative to WT and *ipk1*Δ + *IPK1*, with *ipk1*Δ burdens being marginally higher than those of *ipk1*Δ *kcs1*Δ and *kcs1*Δ. However, the reduction observed for all mutant strains relative to WT was not statistically significant. Only the PP-IP_4_-accumulating *ipk1*Δ mutant strain disseminated to the brain. Our results suggest that the accumulated PP-IP_4_ in *ipk1*Δ is compensating for the absence of PP-IP_5_/IP_7_ to partially “restore” cryptococcal load in the lung and permit dissemination. However, the improvement is not sufficient to restore virulence. Improved growth of the *ipk1*Δ mutant strain in lung tissue and its ability to disseminate to the brain may be attributable in part to its improved capability to utilise alterative carbon sources relative to the other mutants (Fig. 6).

## Discussion

We previously described Arg1, Kcs1 and Asp1 as major IP_3_, IP_6_ and PP-IP_5_/IP_7_ kinases in *C. neoformans*, respectively. We now report that Ipk1 is the major IP_5_ kinase in *C. neoformans*. Deletion of the *IPK1* gene (*ipk1*Δ) abolished the synthesis of IP_6_, and the two inositol pyrophosphates, PP-IP_5_ and (PP)_2_-IP_4_, which are produced by Kcs1 and Asp1, respectively (Fig. 1B). Since PP-IP_5_/IP_7_ is crucial for virulence^23^ and IP_6_ is highly abundant, we expected that the absence of both IP_6_ and PP-IP_5_/IP_7_ in the *ipk1*Δ mutant strain would exacerbate the severity of the phenotypic defect as compared to *kcs1*Δ, which is only deficient in PP-IP_5_/IP_7_. However, this was not the case, since loss of IP_6_ and PP-IP_5_/IP_7_ did not attenuate virulence phenotypes or virulence in animal models to a greater extent than the loss of PP-IP_5_/IP_7_ alone. Thus, IP_6_ has a negligible role in fungal physiology and virulence. In *S. cerevisiae* and *S. pombe*, Ipk1-generated IP_6_ has an essential role in Gle1p-mediated mRNA export at the nuclear pore complex, as mutant strains lacking *IPK1* failed to effectively export mRNA from the nucleus^27^,^28^. So far we have not been able to attribute a role to IP_6_ in *C. neoformans* except that it serves as an essential precursor for the synthesis of PP-IP_5_/IP_7_.

It remains to be determined how PP-IP_5_ exerts its effect in *C. neoformans*. The pleiotropic phenotype of *kcs1*Δ and its transcriptional profile indicate that PP-IP_5_ markedly affects gene expression^29^,^30^,^31^,^32^. PP-IP_5_/IP_7_ might regulate gene expression by interacting directly with components of the transcription regulatory complexes/chromatin remodelling machinery, or by pyrophosphorylating transcriptional regulators to alter their activity^33^,^34^.

**Figure 9:**
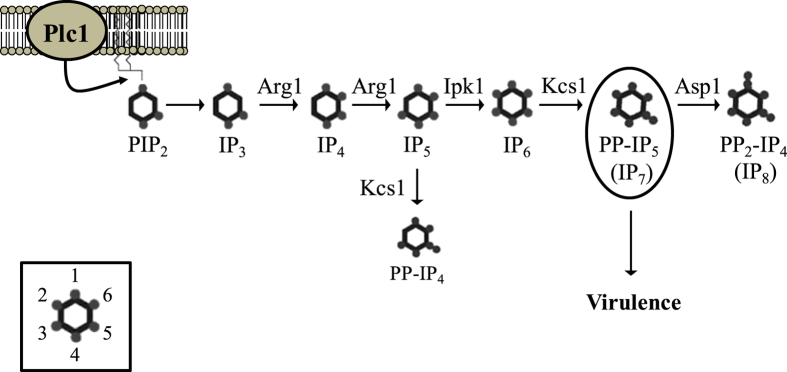
The inositol polyphosphate biosynthesis pathway in *C. neoformans*. Plc1 – produced IP_3_ is sequentially phosphorylated to IP_4_, IP_5_ and IP_6_ by Arg1 and Ipk1. Kcs1 generates PP-IP_4_ and PP-IP_5_/IP_7_ from IP_5_ and IP_6_, respectively. (PP)_2_-IP_4_ is derived from Asp1. The Inset represents the position of the phosphates on the inositol ring. Figure was adapted from^23^.

PP-IP_4_ accumulated in the *ipk1*Δ mutant, but its production was abolished following co-deletion of *KCS1* and *IPK1.* Similar to Kcs1 in *S. cerevisiae*^24^,^35^, our results demonstrate that cryptococcal Kcs1 is the progenitor of PP-IP_4_ when Ipk1 is absent. However, when Ipk1 is present, as in the case of WT and the *kcs1*Δ mutant, PP-IP_4_ is not produced, indicating that Kcs1 acts predominantly as an IP_6_ kinase. Thus, cryptococcal Kcs1 is a dual specificity kinase acting primarily as an IP_6_ kinase to produce PP-IP_5_, and as an IP_5_ kinase producing PP-IP_4_ when IP_6_ is unavailable. However, the physiological significance of PP-IP_4_ production remains to be determined. The predominant roles of Ipk1 and Kcs1 *in vivo* are most likely related to their relative affinities for IP_5_ and IP_6_ and the greater abundance of IP_6_. A model of the complete IP biosynthesis pathway in *C. neoformans* depicting the role of Ipk1 and the new role for Kcs1 is shown in Fig. 9.

None of the mutants in this study (*ipk1*Δ, *kcs1*Δ, *ipk1*Δ *kcs1*Δ) generate PP-IP_5_/IP_7_. However, a comparison of the *ipk1*Δ and *kcs1*Δ phenotypes revealed that the *ipk1*Δ phenotype is more robust than the *kcs1*Δ and *ipk1*Δ *kcs1*Δ phenotypes, particularly in the case of virulence in mice. Although Ipk1 was crucial for virulence, with only 20% of the *ipk1*Δ-infected mice succumbing to infection over a 50 day infection period, no deaths were recorded in the *kcs1*Δ- or *ipk1*Δ *kcs1*Δ-infected groups over the same time period. Furthermore, there was a trend towards *ipk1*Δ producing higher burdens of infection in the lungs and being the only mutant with an ability to disseminate to the brain despite a similar reduction in urease as compared to the other mutants. Urease has been implicated as a factor enabling *C. neoformans* to cause meningoencephalitis^5^.

Nutritional availability in the host lung, glucose in particular, is limited and it is imperative for microbial pathogens to adapt their metabolism accordingly. In agreement with its improved proliferation in the host lung, as compared to Kcs1-deficient mutants, *ipk1*Δ was able to metabolise glycerol, lactate and oleic acid more efficiently and grew significantly better than the other mutants in the presence of these compounds as a sole carbon source. Glycerol, lactate and the unsaturated fatty acid, oleic acid, are incorporated into the tricarboxylic acid (TCA) cycle via different routes. Glycerol is converted to glyceraldehyde 3-phosphate, lactate is oxidised to pyruvate, and oleic acid undergoes β-oxidation to produce acetyl-CoA. Our RNA-seq data demonstrate that genes encoding TCA cycle enzymes are down-regulated in *arg1*Δ, *ipk1*Δ and *kcs1*Δ mutants. However, this down-regulation is less pronounced in *ipk1*Δ than in *arg1*Δ and *kcs1*Δ (Fig. 7B).

A possible explanation for the more robust *ipk1*Δ phenotype (improved metabolism of alternative carbon sources and, to a lesser extent, growth on rich medium) is that PP-IP_4_ can (partially) compensate for the absence of PP-IP_5_/IP_7_ by fulfilling some of its functions. This is supported by the fact that PP-IP_4_ and PP-IP_5_/IP_7_ are structurally similar (see Fig. 9). Both pyrophosphates have a diphosphate at the 5′ position on the inositol ring. The difference between the two species is the absence of a phosphate group at the 2′ position in PP-IP_4_, which is replaced by a hydroxyl group. A similar compensation phenomenon was demonstrated in *S. cerevisiae*, where in the absence of other inositol pyrophosphates, PP-IP_4_ is sufficient to regulate endocytic trafficking and ensure normal vesicular morphology^35^. Due to the minor structural difference, PP-IP_4_ may be able to bind to and/or pyrophosphorylate some, but not all, of the PP-IP_5_/IP_7_ targets. The relative stability of the terminal phosphate on the diphosphate of PP-IP_4_ and its ability to pyrophosphorylate pre-phosphorylated proteins remains to be investigated. Cryptococcal phenotypes uniquely dependent on PP-IP_5_/IP_7_ include cell wall integrity, oxidative/nitrosative stress tolerance, and laccase and urease production.

*C. neoformans* encounters oxidative and nitrosative stress initiated by macrophages during infection. IPs have been reported to act as antioxidants, with IP_6_ being the most potent^36^,^37^. Hawkins *et al.*^37^ determined that the iron (Fe^3+^)-chelating properties of IP_6_ could prevent the formation of toxic reactive oxygen species (ROS). Since iron is also involved in catalysing the production of reactive nitrogen species (RNS)^38^, we assessed the ability of the IPK mutant strains to tolerate oxidative and nitrosative stress by adding hydrogen peroxide (H_2_O_2_) and sodium nitrite (NaNO_2_), respectively, to the YNB growth medium. However, the growth rates of IP_6_-deficient *ipk1*Δ and *ipk1*Δ *kcs1*Δ strains and the IP_6_-producing *kcs1*Δ strain were similar in the presence of either reagent (Fig. 3) suggesting that the absence of IP_6_ is not the crucial factor contributing to the oxidative/nitrosative stress sensitivity of *ipk1*Δ.

Unlike in mammalian cells, IPK enzymes in *C. neoformans* are not redundant and share a low sequence similarity with their mammalian equivalents. Cryptococcal IPK enzymes therefore represent targets for antifungal drug design. *Cn*Ipk1 is only 19.18% identical to the mammalian homolog inositol-pentakisphosphate 2-kinase. *Cn*Kcs1 is 12.65%, 11.69% and 12.09% similar to its three equivalent mammalian kinases, inositol hexakisphosphate kinase 1 isoform 1 (IP6K1), IP6K2 and IP6K3, respectively. Mutant strains lacking *KCS1* and *IPK1* showed increased susceptibility to the azole family of drugs (Supplementary Table S1). This suggests that inhibitors directed against these enzymes, especially Kcs1 due to its crucial role in PP-IP_5_/IP_7_ production, could potentially act synergistically with azoles and improve treatment outcome. Furthermore, IP kinases with low homology to mammalian enzymes are also found in other medically important opportunistic fungal pathogens, including *Candida albicans*, potentially extending the applicability of IPK inhibitors to other fungal pathogens.

## Conclusion

Using gene deletion analysis we have identified Ipk1 as the major IP_5_ kinase in *C. neoformans* and as a major contributor to cryptococcal pathogenicity. However, the means by which Ipk1 contributes to pathogenicity is due to its indirect role in the production of PP-IP_5_/IP_7_, rather than its direct role in producing IP_6_. Our results using double deletion mutants show that the contribution of IP_6_ to pathogenicity is, in fact, relatively insignificant and confirm our previous observation that PP-IP_5_/IP_7_ is the most crucial IP species. We have also demonstrated an additional kinase activity for Kcs1, which is only evident when Ipk1 is absent. This activity involves production of an additional inositol pyrophosphate species, PP-IP_4_. In *ipk1*Δ, PP-IP_4_ function may overlap with PP-IP_5_/IP_7_ to partially restore utilisation of alternative carbon sources, lung infection burdens and dissemination to the CNS, even though *ipk1*Δ infection remains predominantly asymptomatic.

## Methods

### Fungal strains and media

Strains used in this study were wild-type *C. neoformans* var. *grubii* strain H99 (serotype A, MAT*α*) and *ipk1*Δ, *kcs1*Δ, *ipk1*Δ *kcs1*Δ and reconstituted *ipk1*Δ (*ipk1*Δ + *IPK1*) which were all created from WT H99. Strains were routinely grown on YPD (1% yeast extract, 2% peptone and 2% dextrose) or Sabouraud (SAB) agar (1% peptone, 4% glucose, 1.5–2% agar). Urease production was assessed on Christensen’s urea agar (2% urea, 1.5% agar, 0.08% NaH_2_PO_4_.H_2_O, 0.12% Na_2_HPO_4_, 0.1% peptone, 0.1% glucose, 0.5% NaCl, 0.0012% phenol red, pH 6.8). Plates were incubated at 30 °C for 72 hours.

### Creating transgenic strains

The *IPK1* gene deletion construct was made using overlap PCR to join the 5′ flanking region, the neomycin resistance cassette (NEO^R^) and the 3′ flanking region. The flanking regions were amplified from WT H99 genomic DNA and NEO^R^ from plasmid pJAF1 (a gift from Dr John R Perfect, Duke University, Durham, NC, USA)^39^. The deletion construct, which effectively had NEO^R^ in place of the *IPK1* coding region, was then transformed into the WT H99 strain using biolistic transformation^40^. Successful transformants (*ipk1*Δ*::NEO*) whereby the NEO^R^ had replaced the *IPK1* gene by homologous recombination, were selected on YPD agar plates containing 0.5M sorbitol and 100μL/mL geneticin (G418).

To construct the reconstituted strain, *ipk1*Δ + *IPK1*, the *IPK1* gene (with 973 bp of 5′ flank and 622 bp of 3′ flank) was amplified from WT H99 genomic DNA. The hygromycin B^R^ cassette was also amplified by PCR. The two fragments were fused together using overlap PCR. The final fragment was then transformed into the *ipk1*Δ strain by biolistic transformation. Transformants were selected on YPD agar containing 0.5M sorbitol and 350 μL/mL hygromycin B. To create the double gene deletion mutant, *ipk1*Δ *kcs1*Δ, we created a *KCS1* deletion cassette, *kcs1*Δ*::NAT*, using PCR, which was introduced into the *ipk1*Δ strain using biolistic transformation to disrupt *KCS1*. Successful transformants were selected on YPD agar containing 0.5M sorbitol and 100μg/mL nourseothricin. *kcs1*Δ*::NEO* was previously created as described in ref. 23.

### [^3^H]-inositol labelling of inositol poly- (IP) and pyrophosphates (PP-IP)

The protocol used for [^3^H]-inositol labelling of the cryptococcal strains was adapted from^41^. Overnight YPD cultures of WT, *ipk1*Δ, *kcs1*Δ, *ipk1*Δ *kcs1*Δ and *ipk1*Δ + *IPK1* were diluted to OD_600_ = 0.05 in 5 mL YPD containing 10μCi/mL [^3^H] myo-inositol (PerkinElmer) and incubated with shaking at 200 rpm at 30 °C until the culture OD_600_ reached at least 12.8 (18–35 hrs). The cells were pelleted by centrifugation (maximum speed for 10 minutes at 4 °C), washed twice with 1 mL ice-cold YPD and snap-frozen in liquid nitrogen. To extract IP/PP-IPs, the cell pellets were resuspended in extraction buffer (1M HClO_4_, 3mM EDTA, 0.1 mg/mL IP_6_) and homogenised at 4 °C with a MiniBeadbeater-8 cell disrupter (Daintree Scientific, TAS, Australia): 4 × 30 second cycles with 1 minute rest on ice in between cycles. The debris was pelleted by centrifugation (maximum speed for 5 minutes at 4 °C). The pH of IP extracts was neutralised by titration with neutralisation buffer (1M K_2_CO_3_, 3mM EDTA). Samples were then incubated on ice for 2 hours, centrifuged at maximum speed for 10 minutes at 4 °C, and supernatants were collected for anion-exchange HPLC as described in^41^. IP/PP-IP species were identified using the relevant standards. Specifically: ^3^H-IP_6_ was acquired from (PerkinElmer NEN (New England Nuclear)); ^3^H-I(1, 3, 4, 5, 6)P_5_ was prepared using IPMK as previously described^42^; PP-IP_5_/IP_7_ was prepared using IP6K1 as previously described^43^. All the radiolabeled inositol phosphates *in vitro* synthesized were HPLC purified and desalted as described^44^. PP-IP_4_ was purified from ^3^H-inositol radiolabelled *Saccharomyces cerevisiae ipk1*Δ strain^35^.

### Quantification of laccase activity and *LAC1* gene expression

*Laccase activity.*  The assay for quantifying extracellular laccase activity was adapted from^23^ and is based on the oxidation of the laccase substrate, 2, 2′-Azino-bis(3-ethylbenzthiazoline-6-sulfonic acid) (ABTS). All strains were adjusted to OD_600_ = 1 and induced in minimal media without glucose (10μM CuSO_4_, 10 mM MgSO_4_, 29.4 mM KH_2_PO_4_, 13 mM glycine and 3μM thiamine) for 6 hours at 30 °C. Following induction, an assay mixture consisting of cells, distilled H_2_O and 3mM ABTS was incubated at 30 °C over a 120 minute time course, with absorbance readings taken at 15, 30, 60, 90 and 120 mins. At each time point, cells were pelleted by centrifugation, and the absorbance of the supernatant at 436 nm was measured using a spectrophotometer.

*LAC1 gene expression* was measured using SYBR Green qRT-PCR (Corbett Rotor-Gene 6000) with mRNA extracted from cells after a 3 hour induction in minimal media without glucose. *ACT1* was used as a reference gene. Relative quantification was determined using the ΔΔCt method^45^.

### Spot dilution assays

WT, *ipk1*Δ, *kcs1*Δ, *ipk1*Δ *kcs1*Δ and *ipk1*Δ + *IPK1* strains were cultured overnight in YPD broth, serially diluted 10-fold and spotted onto the following medium: YPD agar containing 0.5 mg/mL caffeine or 0.5% Congo red to examine the effect of these cell-wall perturbing agents on growth; minimal media agar supplemented with 1% glucose, 1% glycerol, 1% sodium lactate or 1% oleic acid as the sole carbon source to assess carbon source utilisation; and YNB (pH 4) + 0.5% glucose agar with or without 1mM H_2_O_2_/NaNO_2_ to determine the effect of oxidative/nitrosative stress. Plates were incubated at 30 °C/37 °C for 72–96 hours.

### RNA-seq

The methodology pertaining to RNA-seq is described in^23^. The following equation was used to generate expression values for heat maps: log_2_(FPKM_mutant_/FPKM_WT_). Clustering was performed using Gene Cluster 3 (University of Tokyo, Human Genome Center http://bonsai.hgc.jp/~mdehoon/software/cluster/software.htm#ctv) and the heat maps drawn using Java TreeView (http://jtreeview.sourceforge.net/). Expression data used to generate the heat maps are listed in Supplementary spreadsheet 1. The whole RNA-seq dataset has been deposited in the NCBI GEO database under the accession number GSE78824.

### Murine inhalation model of cryptococcosis

All procedures described were approved and governed by the Sydney West Local Health District Animal Ethics Committee, Department of Animal Care, Westmead Hospital, and all procedures were carried out in accordance with the guidelines and regulations of this institute. Survival and organ burden were conducted using 7-week-old female BALB/c mice obtained from the Animal Resource Centre, Floreat Park, Western Australia. Mice were anaesthetised using isoflurane (in oxygen) delivered via an isoflurane vapouriser attached to a Stinger Small Animal Anaesthetic Machine (Advanced Anaesthesia Specialists).

Groups of 10 mice were inoculated intranasally with WT, *ipk1*Δ, *kcs1*Δ, *ipk1*Δ *kcs1*Δ and *ipk1*Δ + *IPK1* strains (5 × 10^5^ cells/20μl PBS). Viable yeast cells inoculated into the nares were quantified following culture on SAB plates. Mice were observed daily for 50 days for signs of ill-health. Mice were deemed to have succumbed to infection if they had lost 20% of their pre-infection weight and/or if they showed debilitating clinical signs such as respiratory distress, hunching, excessive ruffling and reduced mobility. Sick mice were euthanised by CO_2_ inhalation followed by cervical dislocation. Lungs and brain were removed from 3 mice and assessed for infection burden following homogenisation and quantitative culture as previously described^23^. All healthy mice were also euthanised as above at the end of the study, 50 days post-inoculation, and organ burdens were quantified as above. Differences in survival were analysed with SPSS (Version 20) statistical software, using the Kaplan-Meier method (Mantel-Cox log-rank test), where a *p*-value < 0.001 was considered statistically significant.

## Additional Information

**How to cite this article**: Li, C. *et al.* Identification of a major IP_5_ kinase in *Cryptococcus neoformans* confirms that PP-IP_5_/IP_7_, not IP_6_, is essential for virulence. *Sci. Rep.* **6**, 23927; doi: 10.1038/srep23927 (2016).

## Supplementary Material

Supplementary Information

Supplementary Dataset 1

## Figures and Tables

**Table 1:** Summary of the IPs and PP-IPs present in the various strains used in this study.

	IP_5_	PP-IP_4_	IP_6_	PP-IP_5_/IP_7_
WT H99	+	−	+	+
*ipk1*Δ	+++	+++	−	−
*ipk1*Δ + *IPK1*	+	−	+	+
*kcs1*Δ	+	−	+	−
*ipk1*Δ *kcs1*Δ	+++	−	−	−

+ indicates that the species is present; +++ indicates accumulation of a species; − indicates absence of a species.
