# Supplementary material for: Identification of a major IP5 kinase in Cryptococcus neoformans confirms that PP-IP5/IP7, not IP6, is essential for virulence
Source: Sci Rep. 2016 Apr 1;6:23927. doi: 10.1038/srep23927 (PMC4817067; doi:10.1038/srep23927)
Supplement: Supplementary Information [file srep23927-s1.pdf]

Supplementary Information

Identification of a major IP_5 kinase in *Cryptococcus neoformans* confirms that PP-IP_5/IP_7, not IP_6, is essential for virulence

Cecilia Li,
Sophie Lev,
Adolfo Saiardi,
Desmarini Desmarini,
Tania C. Sorrell and
Julianne T. Djordjevic

Supplementary methods

Antifungal susceptibility testing (Sensititre®)

WT, *ipk1Δ*, *kcs1Δ* and *ipk1Δ kcs1Δ* cells were grown on SAB plates at room temperature for 72 hours, after which several colonies were suspended in sterile distilled water. The density was adjusted to 0.45-0.55 McFarland using the bioMerieux Densichek™ calibrator. 20 µL of the adjusted suspension was added to 11 mL of the YeastOne Inoculum broth, resulting in a final concentration of 1.5-8 x 10^3 CFU/mL. 100µL of inoculum was pipetted into each well of the Sensititre® YeastOne plate (one plate for each strain tested). Plates were incubated at 30°C, 5% CO_2 for 72 hours. Positive growth was indicated by the change in colour of the indicator, alamarBlue®, from blue to pink. Minimum inhibitory concentrations (MICs) were determined from the first blue well (negative growth) and are expressed in µg/mL.

Verifying transgenic strains

PCR To verify that the deletion construct had been introduced at the correct site of integration, PCR was performed across each integration junction with one primer annealing outside the region of integration and the other annealing within the construct. For the reconstituted *IPK1* strain, internal primers within the *IPK1* gene were used to verify the ectopic integration of the *IPK1* gene. A 1kb Plus DNA ladder (Invitrogen) was used.

Spot dilution plate assays were carried out to ensure integration of the antibiotic resistance cassettes into the genome of the mutant strains. WT, *ipk1Δ*, *kcs1Δ*, *ipk1Δ kcs1Δ* and *ipk1Δ + IPK1* strains were 10-fold serially diluted and spotted onto YPD agar containing 100µg/mL NEO, 100µg/mL NAT or 350µg/mL HYG B. Plates were incubated at 30°C for 72 hours.

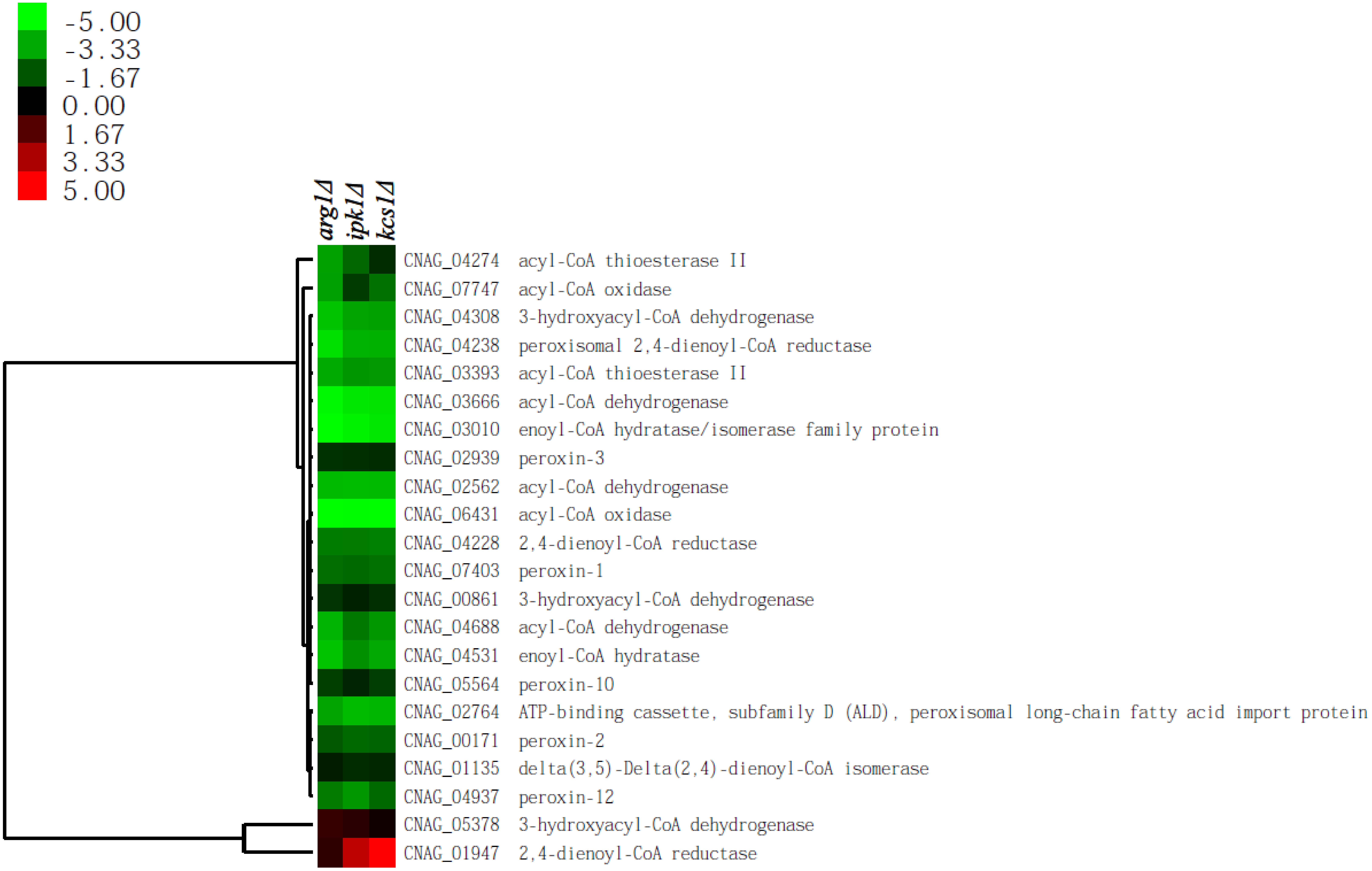

Figure S1: RNA-seq derived heat maps of the IPK mutants, *arg1Δ*, *ipk1Δ* and *kcs1Δ* showing the intensity of expression of genes involved in fatty acid β-oxidation and peroxisomal organisation, relative to WT H99. The colour bar to the left demonstrates the log_2 fold changes from comparison of each mutant to WT (green, down-regulated expression; red, up-regulated expression).

**Strain**	**MIC (μg/mL)**								
	AND	MF	CAS	FC	PZ	VOR	IZ	FZ	AMB
**WT H99**	>8	>8	>8	8	0.25	0.12	0.25	16	0.5
ipk1Δ	>8	>8	>8	2	0.06	0.03	0.06	4	0.25
kcs1Δ	>8	>8	>8	4	0.06	0.03	0.03	4	0.25
ipk1Δ kcs1Δ	>8	>8	>8	2	0.06	0.03	0.03	4	0.5

Table S1: IPK mutants are hypersusceptible to antifungals. Minimal inhibitory concentrations (MICs) were determined using a commercially available colorimetric broth microdilution plate containing the drugs indicated. MICs were recorded as the first well of drug-inhibited growth. AND, Anidulafungin; MF, Micafungin; CAS, Caspofungin; AMB, Amphotericin B; FC, 5-flucytosine; PZ, Posaconazole; VOR, Voriconazole; IZ, Itraconazole; FZ, Fluconazole.

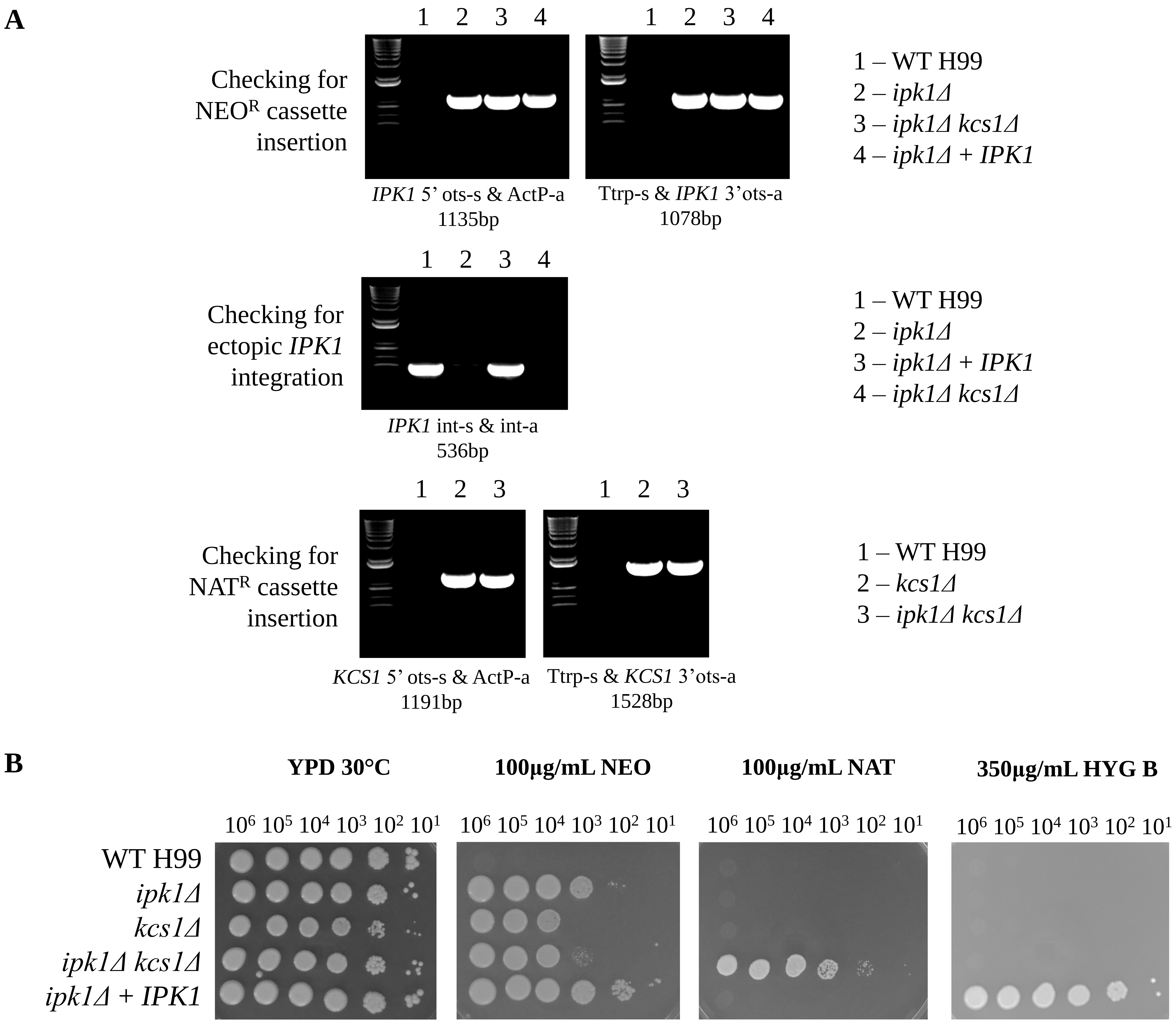

Figure S2: Verifying the targeted integration of *IPK1* and *KCS1* (providing Neo resistance) into the WT genome; ectopic integrations of *IPK1* (providing Hyg B resistance) and *KCS1* (providing Nat resistance) into the *ipk1Δ* genome, respectively.

(A) PCRs Primers used to generate each band are listed in Table S2

(B) Drop assay

Description	Name	Sequence	Name	Sequence
IPK1 deletion construct with NEO; 5’ flank	IPK1 5’-s	AAGATAGAGCTTAGCCGGTTGGAA	IPK1 5’-a	CTCCAGCTCACATCCTCGCAGGGGAGGTTGCAGTTCGATAA
IPK1 deletion construct with NEO; 3’ flank	IPK1 3’-s	CCTCAGGATCTTCATGGCTCCCAAAGGGACCAGATGAAGGA	IPK1 3’-a	ACGCCGACATTGTATCTCTTC
IPK1 deletion verification; 5’ flank	IPK1 ots-s	GCTCCTGCAGAATAGGGTTAGAA	ActP-a	TGTTGTTACCATCATCCTCTCCTC
IPK1 deletion verification; 3’ flank	Ttrp-s	CTACAGACAACAATACCATCCTTCC	IPK1 ots-a	GGTGTACCTAAGCCCGTTGA
IPK1 deletion verification; internal	IPK1 int-s	ATTTCCCACATCGTCCAGAG	IPK1 int-s	GTCGTTAGCCCAAGCAGAAG
KCS1 deletion construct with NAT	KCS1 5’-s	GCTATGCTGAGGAAGCAACG	KCS1 3’-a	GAGCTCGAGGCAGTTGACAG
KCS1 deletion verification; 5’ flank	KCS1 ots-s	AAGGCGAGTTTTGAGATGCT	ActP-a	TGTTGTTACCATCATCCTCTCCTC
KCS1 deletion verification; 3’ flank	Ttrp-s	CTACAGACAACAATACCATCCTTCC	KCS1 ots-a	GTTTCTCTCCCGTGATTCCA
LAC1 qRT-PCR	*LAC1*-s	CAGGGCGGAAGTTGGGTGGT	*LAC1*-a	GTCCAAGACTCGGGGCCCTC

Table S2: Primers used in the study.
